# A Template-Free Microwave Synthesis of One-Dimensional Cu_2_O Nanowires with Desired Photocatalytic Property

**DOI:** 10.3390/ma11101843

**Published:** 2018-09-27

**Authors:** Rui Chen, Zuoshan Wang, Qingqing Zhou, Juan Lu, Min Zheng

**Affiliations:** 1 College of Chemistry, Chemical Engineering and Materials Science, Soochow University, Soochow 215123, China; 20164209195@stu.suda.edu.cn (R.C.); zuoshanwang@suda.edu.cn (Z.W.); qqzhou@stu.suda.edu.cn (Q.Z.); lujuan@suda.edu.cn (J.L.); 2 College of Textile and Clothing Engineering, Soochow University, Soochow 215123, China

**Keywords:** One-dimensional Cu_2_O nanowires, microwave synthesis, growth mechanism

## Abstract

One-dimensional Cu_2_O nanowires were successfully prepared with a template-free microwave synthesis. Neither a surfactant was needed (to induce the growth), nor a long reaction time was required for this method. The structural investigation confirmed the successful preparation of Cu_2_O. The morphology images showed that the radial size of the Cu_2_O nanowires was 10 nm. The possible growth mechanism was hypothesized according to morphology evolution and references. A series of time-dependent experiments indicated that as time increased, Cu_2_O primary particles grew radially into nanowires under microwave energy irradiation. The condition-variable tests revealed that the suitable quantity of NaOH played a vital role in Cu_2_O nanowire formation. The photocatalytic property of the sample was investigated by degradation of methyl orange under the irradiation of visible light at room temperature. Benefiting from its unique large surface area, 4 mg of the prepared catalyst degraded 73% of methyl orange (10 mg L^−1^) in 120 min.

## 1. Introduction

Cu_2_O is a typical narrow bandgap p-type semiconductor, and its band gap is 2.0–2.2 eV [1]. The narrow band structure enables Cu_2_O to utilize visible solar light, the main solar irradiation scattered on the ground. In particular, the electrons on the valence band are excited by visible-light energy to the conduction band, producing active carriers [2]. In this process, the Cu_2_O semiconductor converts the “green” light energy into electronic energy. As a consequence, Cu_2_O is widely studied for its photocatalytic ability, be it either decomposing water into H_2_ or degrading organic pollutants [2,3,4,5,6]. Cu_2_O is also applied in antibacterial applications [7,8,9], in battery electrodes [10,11,12], and sensors [13,14,15] due to its abundance, nontoxicity, and low cost [16]. 

One-dimensional semiconductor materials with many advantages, such as lower material consumption, efficient coupling with sunlight, effective light-to-electricity conversion capabilities, and drastically different physicochemical behaviors from bulk materials have been reported for photovoltaic applications [17,18,19]. Therefore, the synthesis of one-dimensional Cu_2_O is widely studied, with methods such as electrodeposition [20], liquid phase reducing method [21], oxidation method [22] and solvothermal method [23] all being explored. Usually, either a sacrificial hard template (such as a Cu grid) is required to orientate well-aligned Cu_2_O nanowire arrays [24,25,26], or a surfactant soft template (such as sodium dodecyl, o-anisidine, pyrrole, or 2,5-dimethoxyaniline) is needed to guide the radical growth of Cu_2_O [27,28]. Although there is a challenge to achieve the template-free synthesis to form 1D Cu_2_O, a polyol method was recently found to synthesize Cu_2_O nanowire without any template [19]. By only using a precursor of copper acetate and ethylene glycol/diethylene glycol or polyethylene glycol, Cu_2_O nanowire can be formed in hours [29,30,31], in which the polyol is not only applied as the solution but also as a reactant.

Compared with the traditional heating method, microwave irradiation synthesis was reported to possess advantages such as volumetric heating, fast kinetics, homogeneity, selectivity, less energy consumption, and time-saving benefits [32,33]. As a “green” synthesis technique, microwave irradiation is currently paid wide attention for controlling the morphologies of inorganic nanoparticles, including Cu_2_O nanoparticles [32,34,35,36,37,38]. This is because key parameters of a microwave system such as power inputs and heating frequency are expected to have great influence on the structure [39]. However, Cu_2_O nanowires have been scarcely synthesized with a microwave-assisted method. Herein, we apply the microwave-assisted route, combined with the polyol method, to form Cu_2_O nanowires. This synthesis needs no template, strict experimental conditions, or long duration. In this process, ethylene glycol was chosen as reducing agent and solution agent, copper acetate as copper source, sodium hydroxide as a precipitator, and small-size Cu_2_O nanowires were fabricated in minutes without any template.

## 2. Experiment

### 2.1. Materials

Copper acetate (Cu(Ac)_2_H_2_O), sodium hydroxide (NaOH), polyvinyl pyrrolidone (PVP K-30), and ethylene glycol (EG) were purchased from Shanghai Chemical reagent Co., Ltd. (Shanghai, China). All the chemicals were analytical reagents and used as received without further purification and deionized water was used throughout this work.

### 2.2. Preparation

Cu_2_O nanowires were synthesized in a facile microwave-assisted route. The detailed process is as follows: 2 mL 0.1 M Cu(Ac)_2_ was dispersed into 50 mL EG, together with 0.4 g PVP K-30 as a dispersion agent. Then, 2 mL 0.2 M NaOH solution was added dropwise under magnetic stirring. After stirring for a period, the precursor was transferred into a 100 mL three-necked flask for the microwave process. 

For the microwave procedure, the microreactor (XH-100B) was obtained from Beijing Xianghu Technology Development Co. Ltd. (Beijing, China). The above precursor was treated for 8 min with a power of 800 W under microwave energy. Finally, the resulting precipitation was separated through centrifugation and was washed several times by deionized water and ethyl alcohol.

### 2.3. Characterization of the Samples

Crystal structures of the samples were characterized with X-ray diffraction (XRD) on a X’ Pert-Pro MPD X-ray diffractometer (Panalytical, Almelo, Holland) with Cu-Kα radiation (λ = 0.154 nm), scanning from 20° to 80° with a scanning voltage of 40 kV and a scanning current of 100 mA. The prepared powder sample was flattened into the groove of the glass sheet for the XRD test. XRD data were analyzed by the software, Jade (6.0, Materials Data, Inc., New York, NY, USA). Morphology of the samples was observed with transmission electron microscopy (TEM, FEI TecnaiF20, FEI, Hillsboro, AL, USA), scanning electron microscopy (SEM, Hitachi S-4700, Hitachi Limited, Tokyo, Japan) and high-resolution transmission electron microscopy (HRTEM, TecnaiG20, FEI, Hillsboro, AL, USA). The product dispersive ethanol solution was dropped on a silicon slice and dried for SEM test. The product dispersive ethanol solution was dropped on a carbon-filmed copper network for the TEM test. TEM images and Selected Area Electron Diffraction (SADE) patterns were captured by the testing machine, and analyzed by the software Digital Micrograph (3.7.4, Gatan Inc., New York, NY, USA). Their chemical compositions were investigated with energy dispersive X-ray spectroscopy (EDX, Thermo Fisher Scientific, Shanghai, China). Surface area and pore size distribution analyses were taken by Brunauer-Emmett-Teller (BET) method with a Micromeritics Tristar 3020.

### 2.4. Photocatalytic Activity

Photocatalytic evaluations of the prepared one-dimensional Cu_2_O nanowires were performed in a photochemical reaction instrument (XuJiang Electromechanical Plant, Nanjing, China) to degrade methyl orange dye (MO) under visible light irradiation at room temperature. A 500 W xenon lamp (XuJiang Electromechanical Plant, Nanjing, China) equipped with a UV filter (λ > 420 nm) was employed as the irradiation source. The procedure was described as follows: 4 mg of the as-made samples were dispersed into 100 mL MO solution (10 mg L^−1^) under ultrasonic vibration for 5 min. With water flowing around the lamp to keep the temperature steady between 20 °C to 25 °C, the suspension system was irradiated for 90 min. Once the irradiation commenced, 4 mL of the suspension was taken out to remove precipitation at every specific interval. 

A trapping experiment of the active species was done by adding triethanolamine (TEOA) (5 mM), isopropanol (IPA), and ascorbic acid (L-AA) to the MO solution as scavengers of h^+^, ·OH, and ·O_2_^−^, respectively. The concentration of MO was tested after visible light illumination for 120 min. 

## 3. Results and Discussion 

### 3.1. Structure and Morphology

**Figure 1:**
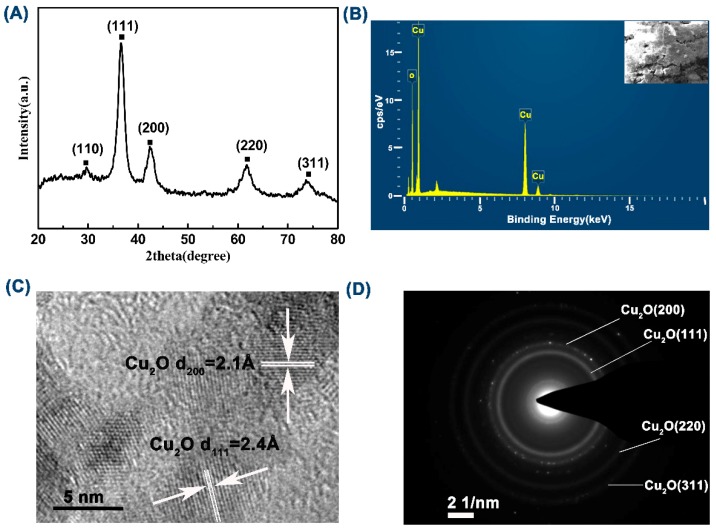
(**A**) X-ray diffraction (XRD) pattern of the prepared Cu_2_O; (**B**) EDX map of the prepared Cu_2_O; (**C**) high-resolution transmission electron microscopy (HRTEM) image of the prepared Cu_2_O; (**D**) selected area electron diffraction image of the prepared Cu_2_O.

In order to confirm the phase composition of the prepared sample, the structure was characterized by XRD analysis, see Figure 1A. The diffraction peaks with 2θ value of 29.5°, 36.4°, 42.3°, 61.5°, 73.6°, and 77.8° corresponded to the (110), (111), (200), (220), (311), and (222) crystal planes of the face-centered cubic Cu_2_O phase, respectively. The space group was Pn-3m (224), a = 4.270, b = 4.270, and c = 4.270, (JCPS#99-0041). To further validate the composition of the prepared samples, the element distribution of the sample was checked by EDS analysis, see Figure 1B. As the graph showed, the sample included two elements, Cu and O. In particular, the peak at around 2.2 keV belonged to Au, sprayed onto the sample to increase the conductivity, and the weak peak to the left of the O peak belonged to adventitious carbon from the sample preparation or the EDS instrument itself [40].

The HRTEM investigation, see Figure 1C, confirmed that the basal spacings of nanowires was 2.4 Å and 2.1 Å, which were consistent with the spacing of the (111) and (200) crystal plane of Cu_2_O (JCPS#99-0041). In the selected-area electron diffraction image, see Figure 1D, four diffraction rings with lattice features corresponded to the (200), (111), (220), and (311) planes. Figure 1C also revealed that the prepared Cu_2_O nanowire was not composed of radially-grown mono-crystal but was constituted by aggregates of nanocrystals, which were around 5 nm in dimeter. In conclusion, the above analyses supported each other and confirmed the successful preparation of the Cu_2_O crystalline structure.

**Figure 2:**
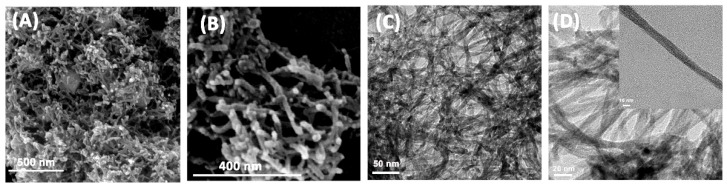
Scanning electron microscopy (SEM) (**A**,**B**) and transmission electron microscopy (TEM) (**C**,**D**) images of the prepared Cu_2_O one-dimensional nanowires.

To further investigate the morphologies and microstructures of the as-prepared samples, SEM and TEM images of different magnifications were taken. As shown in Figure 2, the prepared Cu_2_O appeared to have a one-dimensional nanowire structure, of which the diameter was around 10 nm. The nanowires cross and overlap each other with large slits in between. The high-resolution image showed the nanowires were well-arranged.

**Figure 3:**
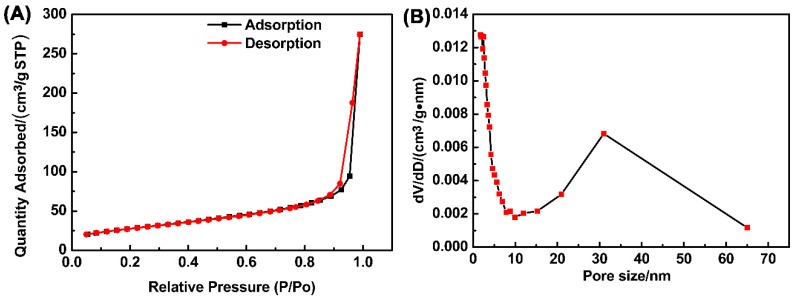
Nitrogen absorption-desorption isotherms of Cu_2_O (**A**); Pore size distributions of Cu_2_O (**B**).

Compared with bulk material, one-dimensional material tends to possess a larger surface area. To characterize the surface area of the product, nitrogen absorption-desorption isotherms, see Figure 3A, were tested using the Brunauer-Emmett-Teller (BET) method, which suggested the specific surface area of the prepared one-dimensional Cu_2_O nanowires was 99.98 m^2^ g^−1^. As shown in Figure 3A, the prepared one-dimensional Cu_2_O exhibits type-IV isotherms with H3-type hysteresis loops. The absorption isotherm rose abruptly under high pressure (0.9–1.0) due to mass capillary condensation. An H3-type hysteresis loop suggested that most pores are large mesopores or macropores, attributed to the slits among the nanowires. Large surface areas provide more active sites for photocatalysis. The pore size distribution of the samples was also obtained from the Barret-Joyner-Halenda (BJH) desorption isotherm. As Figure 3B shows, the sample displayed a major large mesopores and macropores distribution, which was in accordance with the absorption-desorption isotherms.

### 3.2. The Mechanism Analysis 

**Figure 4:**
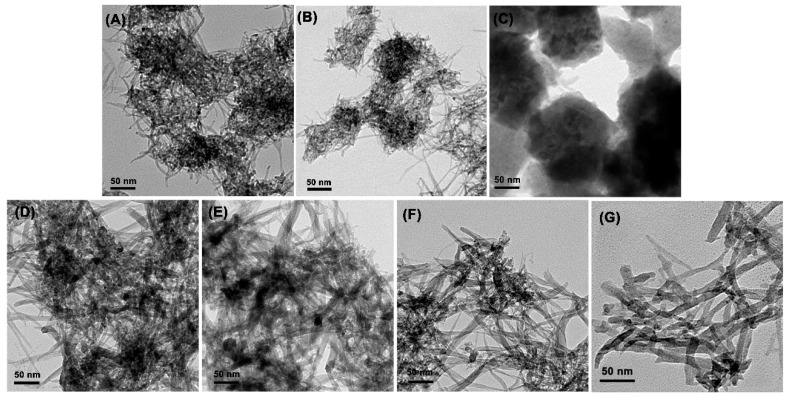
TEM images of the prepared Cu_2_O on different conditions: without polyvinyl pyrrolidone (PVP) (**A**); NaOH (0.2 M) increased to 3 mL (**B**) and 4 mL (**C**); different microwaving times: 2 min (**C**), 4 min (**D**), 6 min (**E**), 8 min (**F**); possible formation route of the prepared one-dimensional Cu_2_O.

To make the influence of each component on the Cu_2_O nanowire clear, control experiments were performed. It was found that the morphology of the product is influenced greatly by the quantity of sodium hydroxide. Firstly, moving NaOH out of this system, there was no precipitate after the microwave process, so NaOH worked as the precipitate agent as in the other reported Cu_2_O synthesis [36,38,41]. According to the report, Cu(OH)_2_ is promoted as soon as NaOH is added into the reaction medium, so the quantity of NaOH in the preparation influences the morphology of metallic oxides in some way. In this experiment, the morphology of Cu_2_O turned from one-dimensional nanowires into three-dimensional bulk when the Cu^2+^/2OH^−^ ratio surpassed 1:1 to 2:3, see Figure 4B, and 1:2, see Figure 4C, in this process. In fact, it was observed that Cu_2_O nanowires tend to agglomerate into bulk as long as the Cu^2+^/2OH^−^ ratio surpassed 1:1. Thus, the quantity of NaOH played a key factor in this process. Secondly, getting rid of PVP, the TEM image indicated that the morphology of the sample was also a one-dimensional material but in severe aggregation, see Figure 4A. As a result, PVP was supposed to work as the dispersing agent, which restricted the particles from aggregation in this preparation, although it was often used to induce the growth of one-dimensional materials [21]. EG played three roles in this system. First, it worked as a solvent, which owns a comparatively high loss tangent (1.17) [42] and can couple with the microwave energy more efficiently. Second, it acted as a reductant. Third, it promoted the formation of small-sized 1D Cu_2_O by modifying the mobility of the primary particles in suspension as well as its effective collision rates [42].

In order to figure out the growth process of the one-dimensional Cu_2_O nanowires, TEM images of samples at different reaction times (2, 4, and 6 min) were taken, see Figure 4D–G. It could be observed that as time increased, Cu_2_O primary particles disappeared, then grew into nanowires in an orderly fashion. The possible reaction process is described as follows:

Cu^2+^ + 2OH^−^ → Cu(OH)_2_, (1)

Cu(OH)_2_ + OHCH_2_CH_2_OH → Cu(OH) + OHCH_2_COOH + 3H^+^, (2)

2Cu(OH) → Cu_2_O + H_2_O, (3)

**Figure 5:**
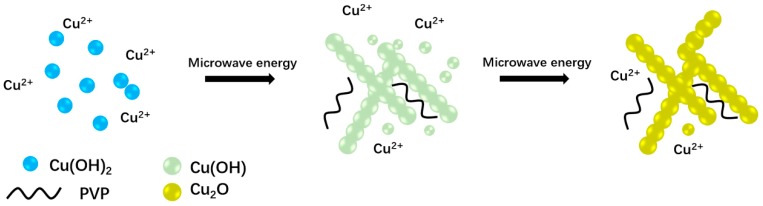
A Possible formation route of the one-dimensional Cu_2_O.

Based on the control experiment and the images in progress above, the growth process can be hypothesized as follows: [31] Firstly, at room temperature, Cu^2+^ hydrolyzed into Cu(OH)_2_ nuclei as soon as NaOH was added, which is accompanied by a color change from transparent light blue to semitransparent dark blue [36,38,41]. Since the NaOH was added dropwise, and the viscosity of the ethylene glycol solution was much higher than water, the combination rate was slow, see Reaction (1). Secondly, Cu(OH)_2_ was reduced to Cu(OH) by EG under microwave energy, see Reaction (2). The reducing ability of EG is closely related to temperature, and increases at higher temperatures, which means that Cu^2+^ reduction reaction was only possible under high temperature [31]. In this process, if the quantity of OH^−^ in Reaction (1) is in excess, the redundant OH^−^ would neutralize H^+^ in Reaction (2), accelerating Reaction (2), improving the growth of particles. Also, excess OH^−^ would easily coordinate with the Cu(OH)_2_ nucleus, and Cu(OH)_2_ nucleus complexation may lead to rapid growth of Cu_2_O during microwave irradiation. These two points explained why the morphology of Cu_2_O turned from one-dimensional nanowires into three-dimensional bulk when the quantity of sodium hydroxide was in excess, see Figure 4B,C. Finally, through a dehydrogenation reaction, Cu(OH) was converted into Cu_2_O. In conclusion, two important conditions which contributed to the one-dimensional growth of Cu_2_O are the suitable quantity of NaOH and the microwave energy. On the one hand, a low concentration of NaOH slowed the reaction and prevented the instant growth, on the other hand, a high microwave energy motivated the particles to grow via high-velocity collisions. Figure 5 illustrates the possible formation route of the prepared one-dimensional Cu_2_O.

### 3.3. Photocatalytic Activity

**Figure 6:**
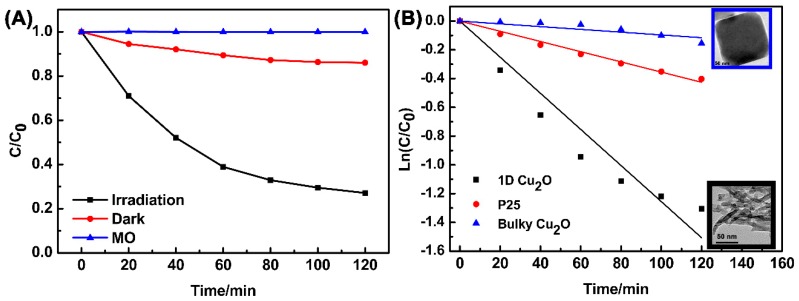
Photocatalytic degradation of methyl orange (MO) versus visible light irradiation duration by Cu_2_O (**A**); the kinetics of MO degradation using various photocatalysts (**B**).

The degradation of MO was investigated as a model reaction to evaluate the photocatalytic activity of synthesized catalysts under visible light (420 nm < λ < 780 nm) irradiation. The degradation rate was calculated according to the following formula: η = C C_0_^−1^, where C and C_0_ stand for reaction and initial concentrations of MO, As Figure 6A showed, the prepared one-dimensional Cu_2_O nanowires removed 73% of the MO in 120 min. A dark experiment of prepared one-dimensional Cu_2_O nanowires was also done to determine the amount of absorption from degradation. Figure 6A suggested that in dark experiments, only 14% of the MO was absorbed, which confirmed the effect of photocatalysis. The blank experiment showed that, without the addition of a photocatalyst, MO dye molecule could not be degraded under visible light irradiation. All in all, most of the dyestuff was decomposed by prepared one-dimensional Cu_2_O nanowires under visible light irradiation for 120 min with only 4 mg of the prepared catalyst. 

To further study the photocatalytic activity of the as-prepared catalyst, the Langmuir-Hinshelwood model was applied. The pseudo-first-order kinetic equation: −ln(C C_0_^−1^) = *kt* was used to describe the reaction kinetics, where *k* is the kinetic constant. The higher rate constant *k* indicates the faster degradation rate [43]. As shown in Figure 6B, the MO photodegradation was in accordance with pseudo-first-order kinetics. The kinetic constants (*k*) and regression coefficients (R^2^) listed in Table 1 were obtained from the simulated straight lines in the plot of ln(C C_0_^−1^) versus time. The prepared Cu_2_O presented much better photocatalytic performance than commercial P25 and bulky Cu_2_O, which were 3.5 and 13 times greater, respectively. This could possibly be ascribed to the visible light response and large active site area of the prepared Cu_2_O.

Generally, photocatalytic degradation starts from the generation of photogenerated electron-hole pairs under light irradiation. The electrons in the valence band can be excited to the conductive band, leaving holes in the valence band. The free electrons in the conductive band can be scavenged by O_2_ and transformed into active ·O_2_^−^, and the holes in the valence band can react with H_2_O and form ·OH [44], as shown in the following reactions:

e^−^ + O_2_ →·O_2_^−^, (4)

h^+^ + H_2_O → H^+^ + ·OH, (5)

**Figure 7:**
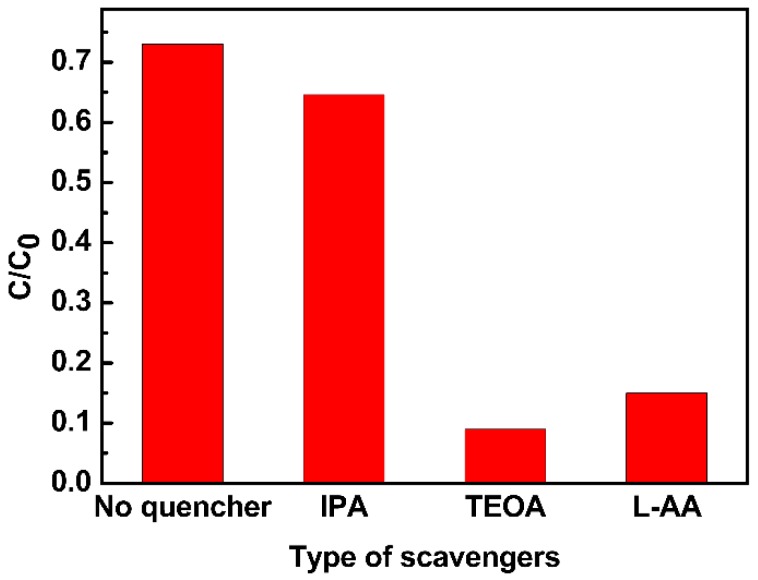
Trapping experiment of the active species.

Several reactive intermediate species such as ·O_2_^−^, and ·OH, together with h^+^, are capable of degrading in photocatalytic processes. The role the reactive species plays in the photocatalytic process was explored by a trapping experiment. In the experiment, IPA, TEOA, and L-AA were used as typical scavengers of ·OH, h^+^ and ·O_2_^−^, respectively [45]. As Figure 7 shows, the degradation rates of MO reduced from 73.0% to 64.6%, 9%, and 15%, respectively. In conclusion, h^+^ and ·O_2_^−^ species made the biggest contributions to MO degradation [45]. This result provides us with an insight to improve the photocatalytic ability of the product by suppressing h^+^ converting to ·OH. To accomplish this, an acidic medium should be enough to suppress Reaction (5) and save the holes for degradation. In the meantime, making a composite using a second component in addition to Cu_2_O for electron and hole separation is a typical route for photocatalytic improvement.

As for the reusability of the prepared Cu_2_O nanowires, we found that the degradation reduced greatly after recycling, and part of Cu_2_O was oxidized to CuO, according to the XRD result. We assume it was because the prepared small-sized Cu_2_O nanowires were unstable in water after a long period of irradiation. Therefore, further research is needed to protect the Cu_2_O product for the purpose of recycling and to improve its photocatalytic properties.

## 4. Conclusions

In this work, we prepared one-dimensional Cu_2_O nanowires in an efficient and template-free microwave method. The process is characterized by the short time required and the fact that no inducing agent is used. The successful preparation of Cu_2_O was confirmed by XRD, EDS, and HRTEM analyses. The possible synthetic mechanism was studied and hypothesized. The quantity of NaOH and the microwave energy were confirmed to work synergistically toward the formation of one-dimensional Cu_2_O nanowires. The preferred photocatalytic ability of the prepared sample was examined by degrading MO under visible light at room temperature. The result showed 73% of the MO was removed under visible light irradiation for 120 min with only 4 mg of the prepared catalyst. However, the reuse stability and other applications of the product need further improvement. This research provides a novel method for the preparation of one-dimensional Cu_2_O nanowires. We believe this microwave-assisted preparation may provide a green and new ideal for one-dimensional material synthesis.

## Figures and Tables

**Table 1:** Degradation parameters of kinetic constant (*k*, min^−1^) and R^2^ using different photocatalysts under visible light irradiation.

Sample	Kinetic Constant (*k*, min^−1^)	R^2^
One-dimensional Cu_2_O	1.26 × 10^−2^	0.976
P25	3.55 × 10^−3^	0.995
Bulky Cu_2_O	9.64 × 10^−4^	0.877
